# The Impact of Peeling on Highland Barley’s Digestive Properties: In Vitro and In Vivo Insights

**DOI:** 10.3390/foods14101686

**Published:** 2025-05-09

**Authors:** Yuting Yan, Xin Gao, Yi Zhang, Fan Xie, Lianzhong Ai

**Affiliations:** 1 Shanghai Engineering Research Center of Food Microbiology, School of Health Science and Engineering, University of Shanghai for Science and Technology, Shanghai 200093, China; 2 Crop Breeding & Cultivation Research Institute, Shanghai Academy of Agricultural Sciences, Shanghai 201403, China

**Keywords:** highland barley, digestive properties, peeling, gut microbiota, glycemic index

## Abstract

Highland barley is a low-glycemic-index grain, and its slow-digesting starch can help delay or prevent the onset and progression of type Ⅱ diabetes. Peeling processes can alter the composition of highland barley, potentially changing its digestive properties. This study explored how changes in nutritional components due to different peeling times (zero, one, two, and three times) affected the digestion and absorption of barley during the gastric and intestinal phases and the utilization of undigested substrates at the distal end of the digestive tract, as well as their impact on fasting blood glucose regulation. The findings indicated that highland barley with fewer peeling times, which retained its outer layer that is rich in dietary fiber, protein, and polyphenols, delayed starch digestion and exhibited better hypoglycemic effects. Compared to unpeeled highland barley, the starch digestion rates of highland barley with one, two, and three peeling times increased by 2.82%, 18.62%, and 26.43% (*p* < 0.05). Based on microstructure, at the same enzymatic digestion time, starch with fewer peeling times retained a more intact granule structure. In mice with dysregulated glucose and lipid metabolism induced by the HFD/STZ method, highland barley with fewer peeling times exhibited a stronger hypoglycemic effect (6.13 mmol/L and 6.07 mmol/L). Additionally, the highland barley dietary intervention improved the gut microbiota composition in these mice, restoring the *Firmicutes/Bacteroidetes* ratio balance and enriching various probiotics, such as *Akkermansia* and *Lactobacillus*. Furthermore, this effect was inversely proportional to the number of peeling times.

## 1. Introduction

Highland barley is native to the Tibetan Plateau and distributed across regions like Tibet, Qinghai, and Gansu in China [1]. It is known for its high dietary fiber (15.01–21.45%), high protein (6.35–23.4%), high vitamin content (vitamin B 3.89–10.84 mg/100 g and vitamin E 0.3–0.8 mg/100 g), low fat (1.18–3.09%), and low sugar (GI 39.4–47.5) composition [2]. Highland barley is distinguished from hulled barley by its lower content of starch, but higher content of protein, plant sterols, and minerals. Additionally, it is rich in bioactive compounds like soluble dietary fiber (β-glucan) and polyphenols, which promote health by improving lipid metabolism and insulin sensitivity [3]. These health benefits are largely attributed to the synergistic regulation of starch digestion by these endogenous non-starch components, which is a crucial aspect of their overall impact on metabolic health. Specifically, the unique composition ratio of highland barley helps slow starch digestion, thereby mitigating postprandial glucose surges. This offers significant benefits in preventing and managing metabolic disorders like type Ⅱ diabetes and obesity [4,5,6].

Highland barley has recently gained significant attention in nutritional research and emerged as a key ingredient in the development of functional foods. Specifically, highland barley can delay starch digestion and absorption, helping to mitigate postprandial blood glucose elevation and improving metabolic responses in the upper gastrointestinal tract [7]. Additionally, its abundant dietary fiber and polyphenolic compounds undergo microbial fermentation in the colon, regulating gut microbiota composition and promoting intestinal health [8,9]. As a result, highland barley exerts effects at different stages of the digestive process, influencing the host’s metabolic health.

In the commercial production of highland barley, the peeling process is a crucial pre-treatment step that directly affects the retention of the grain’s structural layers and bioactive components. The main purpose of peeling is to remove the tough, indigestible outer husk, improving the grain’s edibility. However, excessive peeling may lead to a loss of bioactive compounds like dietary fiber and polyphenols, which are concentrated in the outer layers. Studies have shown that peeling causes changes [10], including increased starch content, reduced dietary fiber, and lower polyphenol levels [11].

Studies have demonstrated that peeling 10% of highland barley positively affects blood glucose [12], tissue pathology, and gut microbiota in mice on a high-fat diet, but no comparison was made between different peeling times. Similar findings were reported for oats and barley. Zhou et al. showed that oats enhanced insulin sensitivity and plasma cholesterol levels in mice compared to low-bran oats [13], suggesting that the increased β-glucan content (by 1.4%) may explain the observed differences. Roasting barley flour reduced the molecular weight of β-glucan, increased resistant and damaged starch, and showed better effects on lipid metabolism compared to untreated barley flour [14]. However, research on how varying peeling times influence the digestive and functional characteristics of highland barley is currently lacking.

This study investigates the impact of zero, one, two, and three peeling treatments on the nutritional composition and digestive characteristics of highland barley. We compared the changes in the nutritional components of highland barley with varying peeling times and investigated the mechanisms behind these changes through a study of viscoelasticity and microstructure during digestion. We also examined how peeling affects the hypoglycemic activity of highland barley in mice with dysregulated glucose and lipid metabolism through a highland barley diet. The findings offer scientific evidence for its industrial application and role in metabolic disease management.

## 2. Materials and Methods

### 2.1. Materials

Highland barley, variety Kunlun 15, was obtained from Qinghai Shangkang Biotechnology Co., Ltd. (Xining, Qinghai, China). The grains were cleaned and sorted, followed by peeling using a rice milling machine from Hunan Chenzhou Machinery Company (Chenzhou, Hunan, China). The machine was set to a fixed pressure of 50 Pascals, determined based on the type of grain. The highland barley was considered to have undergone one peeling after the first treatment, and after the second treatment, it was considered to have undergone two peelings, and so on, resulting in highland barley with different peeling times (PTHB0, PTHB1, PTHB2, and PTHB3). The highland barley seeds were coarsely ground using a universal grinder (BAIJIE 1000A, Huzhou, Zhejiang, China) and then passed through an 80-mesh sieve. The ground highland barley flour was repeatedly sieved to obtain whole highland barley flour.

Total starch, dietary fiber, and β-glucan kits were sourced from Megazyme International Ireland (Bray Co., Wicklow, Ireland). The glucose oxidase (GLU) assay kit came from Nanjing Jiancheng Bioengineering Institute (Nanjing, Jiangsu, China). Pepsin (S10027, ≥30,000 units/g), pancreatin (S10031, trypsin ≥ 4000 units/g, α-amylase ≥ 7000 units, lipase ≥ 4000 units/g), and amyloglucosidase (S10017, ≥100 units/mg) were supplied by Shanghai Yuanye Bio-Technology Co. (Shanghai, China).

### 2.2. Nutritional Components

Protein and lipid contents were determined using AOAC methods 979.06 (2000) and 920.39 (2000) [15]. Total starch, dietary fiber, and β-glucan were quantified using commercial kits.

#### 2.2.1. Total Starch

We mixed 100 mg of the highland barley flour with 0.2 mL of 80% ethanol. Then, added 2 mL of cold 1.7 M sodium hydroxide solution and incubated the solution in an ice-water bath for 15 min. Then, thermostable α-amylase and amyloglucosidase were added, and incubated at 50 °C for 30 min. After cooling, the solution was centrifuged (6010× *g*, 5 min). The starch content was determined using the GOPOD method by measuring the absorbance at 510 nm.

#### 2.2.2. Dietary Fiber

We weighed 1 g of the highland barley flour and added 50 mL of phosphate buffer (pH 6.0). Then, 50 µL of α-amylase, 100 µL of protease, and 200 µL of amyloglucosidase were sequentially added for digestion. Then, 280 mL of pre-heated 95% ethanol (at 60 °C) was added and the precipitation was left to form at room temperature for 60 min. The residue was washed and dried in a vacuum oven at 70 °C. The dietary fiber content was determined based on the residue.

#### 2.2.3. β-Glucan

We weighed 100 mg of the highland barley flour and added 0.2 mL of 50% ethanol and 4.0 mL of 20 mM sodium phosphate buffer (pH 6.5). The mixture was incubated in a boiling water bath for 2 min. Then, 0.2 mL of lichenase solution was added and incubated at 50 °C for 1 h. The solution was centrifuged (1000× *g*, 10 min) and 0.1 mL of the supernatant was transferred and added to 0.1 mL of β-glucosidase. The absorbance was measured at 510 nm using the GOPOD method to calculate the β-glucan content.

### 2.3. Extraction of Polyphenols

The polyphenols in highland barley were extracted and quantified based on the approach of Wang et al. [16]. A 1.00 g highland barley flour with varying peeling times was mixed with 20 mL of precooled 80% acetone. After ultrasonic extraction (300 W, 30 min) and centrifugation (6010× *g*, 15 min), the supernatant was collected. The extraction was repeated three times, and the combined supernatants were concentrated to a final volume of 10 mL. The extract was stored at −20 °C in the dark.

### 2.4. Total Phenol Content

The total polyphenol content was determined by the Folin–Ciocalteu method with slight modifications. Specifically, an aliquot of the polyphenol extract was mixed with 400 μL of distilled water and 100 μL of Folin reagent, vortexed, and incubated in the dark for 6 min. Next, 1 mL of 7% Na_2_CO_3_ solution and 800 μL of distilled water were added, followed by mixing and incubated in the dark for 90 min. The polyphenol content was given in mg of gallic acid equivalent (GAE) per 100 g of dry matter. Absorbance was recorded at 760 nm.

### 2.5. Antioxidant Activity Analysis

The antioxidant ability was evaluated using the modified method of Chen et al. [17].

#### 2.5.1. DPPH Radical Scavenging Capacity

Samples were mixed with 2 mL DPPH ethanol solution (0.2 mmol/L) and incubated in the dark for 30 min. By evaluating the absorbance of the sample against that of the sample-free solution, the DPPH radical scavenging capacity was determined, with the results expressed as a percentage (%). Absorbance was measured at 517 nm.

#### 2.5.2. ABTS Radical Scavenging Capacity

The ABTS working solution was made by combining equal volumes of 7.4 mmol/L ABTS solution and 2.6 mmol/L potassium persulfate solution, followed by a 12 h reaction in the dark at 25 °C. 1 mL of the ABTS stock solution was diluted with 40 mL methanol to obtain the working solution. Samples were then mixed with 2.85 mL of the ABTS working solution and incubated in the dark for 20 min. By evaluating the absorbance of the sample against that of the sample-free solution, the ABTS radical scavenging capacity was determined, with results expressed as a percentage (%). Absorbance was measured at 734 nm.

#### 2.5.3. Hydroxyl Radical Scavenging Capacity

Samples were combined with 0.5 mL of 5 mmol/L ferrous sulfate solution, 0.5 mL of 5 mmol/L hydrogen peroxide solution, and 0.5 mL of salicylic acid ethanol solution. The mixture was then incubated in a 37 °C water bath for 30 min. By evaluating the absorbance of the sample against that of the sample-free solution, the hydroxyl radical scavenging capacity was determined, with results expressed as a percentage (%). Absorbance was measured at 510 nm.

### 2.6. In Vitro Starch Digestibility Analysis

The starch digestibility of highland barley with different peeling times was assessed using the method outlined by Yang et al. [18]. A 600 mg sample was mixed with 10 mL of sodium acetate buffer (0.2 mol/L, pH 5.2), gelatinized by boiling for 30 min, and incubated at 37 °C in a shaking incubator (200 r/min, 10 min). A pepsin solution (10 mL, 5 mg/L) was added, followed by incubation for 30 min. Afterward, 5 mL of sodium acetate buffer and 5 mL of a pancreatin-amylase mixture were added, and incubated at 37 °C in a shaking incubator (200 r/min, 180 min).

At designated time points (0, 20, 30, 40, 50, 60, 90, 120, 150, and 180 min), the enzymatic reactions were terminated by heating for 10 min, and starch hydrolysis was quantified using the glucose oxidase assay. The starch hydrolysis rate (%), and the contents of rapidly digestible starch (*RDS*), slowly digestible starch (*SDS*), and resistant starch (*RS*) were determined. The starch digestion rate constant (*k*) and final hydrolysis extent (*C_∞_*) were calculated using a nonlinear regression model.

The glycemic index (*GI*) was estimated from the hydrolysis index (*HI*), computed as the ratio of the sample’s hydrolysis area under the curve (*AUC*) to that of the reference sample [19].

*Starch hydrolysis rate* (%) = (*Gt* × 0.9)/*TS* × 100% (1)

where *Gt* represents the glucose released during the time interval of *t* (0–180 min); *TS* represents the total starch.

*RDS* (%) = ((*G20* − *G0*) × 0.9)/*TS* × 100% (2)

*SDS* (%) = ((*G120* − *G20*) × 0.9)/*TS* × 100% (3)

*RS* (%) = ((*TS* − *RDS* − *SDS*) × 0.9)/*TS* × 100% (4)

where *RDS* refers to the rapidly digestible starch, *SDS* to slowly digestible starch, *RS* to the resistant starch, *G20* to the glucose liberated (mg) during the 0–20 min, and *G120* to the glucose liberated (mg) during 20–120 min.

*Ct* = *C*_∞_ [1 − *e* − *kt*], (5)

*GI* = 0.549 × *HI* + 39.71, (6)

where *GI* is the glycemic index and *HI* is the hydrolysis index.

### 2.7. Rheological Simulation of Highland Barley Digestion

The digestion reaction mixture of highland barley samples with different peeling treatments was subjected to a rheological time sweep on a rheometer (Discovery, HR-3, TA Instrument Inc., New Castle, DE, USA), maintained at a constant temperature of 37 °C for 180 min. The experimental conditions were set as follows: strain: 1.0%; angular frequency: 10.0 rad/s.

### 2.8. Microstructure Analysis

The internal structure of the digested highland barley mixture was examined using scanning electron microscopy (ZEISS GeminiSEM 300, Oberkochen, Baden-Württemberg, DE). Samples were attached to conductive adhesive and gold-coated by sputtering (45 s, 10 mA, Quorum SC7620, Oxford, Oxfordshire, UK), and analyzed under SEM. Morphological imaging was performed at 3 kV, and energy spectrum mapping was carried out at 15 kV using an SE2 secondary electron detector. Images were captured at 3000× magnification.

### 2.9. Animal Experiment

Six-week-old male C57BL/6 mice were sourced from Shanghai Bikai Keyi Biotechnology Co., Ltd., Shanghai, China. The mice were kept at 24 °C with a 12 h light/dark cycle and had ad libitum access to food and water. A dysregulated glucose and lipid metabolism model was established in mice using the HFD/STZ method [20]. After one week of acclimatization, all mice, except for the normal control (NC) group, were fed a high-fat diet (HFD, 60% energy from fat) for 4 weeks. After overnight fasting, mice were injected intraperitoneally with STZ (80 mg/kg, citrate buffer, pH 4.5).

Mice exhibiting >20% weight gain over controls and significantly elevated fasting blood glucose (FBG) were selected for dietary intervention with highland barley. Groups included: The HFD group continued to consume a high-fat diet. The PTHB0 group was administered 100% highland barley with no peeling. The PTHB1 group was administered 100% highland barley with one peeling. The PTHB2 group was administered 100% highland barley with two peelings. The PTHB3 group was administered 100% highland barley with three peelings. After the dietary intervention, fasting blood glucose levels were measured weekly for a duration of 4 weeks. Fasting blood glucose was measured three days post-injection via tail vein sampling (ACCU-CHEK Performa, Corydon, ID, USA).

### 2.10. Gut Microbiota Analysis by 16S rDNA

Gut microbiota composition was analyzed via 16S rDNA sequencing. Genomic DNA was isolated with the E.Z.N.A.^®^ Soil DNA Kit (Omega Bio-tek, Norcross, GA, USA), and the V3-V4 region of the 16S rRNA gene was amplified using the specific primers 338F (5′-ACTCCTACGGGAGGCAGCAG-3′) and 806R (5′-GGACTACHVGGGTWTCTAAT-3′), incorporating barcode sequences. Purified PCR products were used for library construction and sequenced on the Illumina PE300/PE250 platform (Shanghai Meiji Biological Medicine Technology Co., Ltd., Shanghai, China).

### 2.11. Statistical Analysis

All experimental data are presented as mean ± standard deviation (SD), and statistical analysis was performed using one-way ANOVA followed by Duncan’s post hoc test in SPSS software (IBM SPSS Statistics 27.0 software, Chicago, IL, USA).

## 3. Results and Discussion

### 3.1. Nutritional Composition

**Figure 1:**
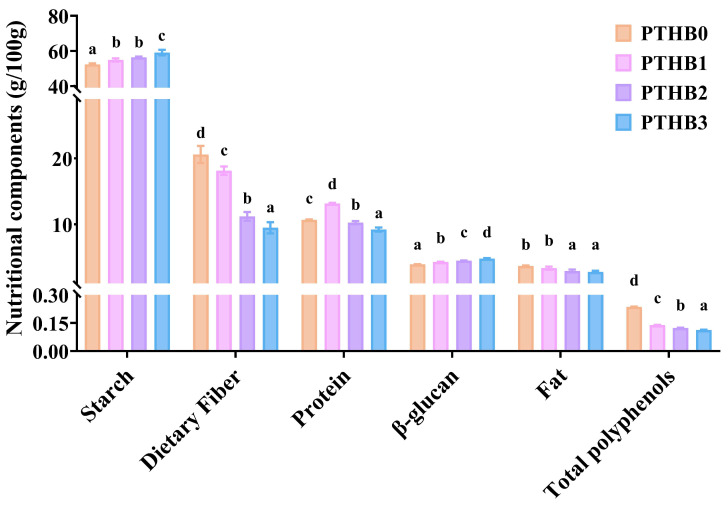
Nutritional components. Unpeeled highland barley (PTHB0), Highland barley with one peeling (PTHB1), Highland barley with two peelings (PTHB2), Highland barley with three peelings (PTHB3). The different letters (a, b, c, d) in the figure indicate significant differences between groups (*p* < 0.05).

According to Figure 1, as the peeling time increased, the contents of polyphenols, fat, and protein showed a decreasing trend, while the starch and β-glucan levels increased. Specifically, the dietary fiber content decreased from (20.60%, *w*/*w*) in PTHB0 to (18.14%, *w*/*w*), (11.20%, *w*/*w*), and (9.47%, *w*/*w*) in PTHB1, PTHB2, and PTHB3, respectively. The most pronounced reduction in dietary fiber occurred after the second peeling, leading to a 45.65% decrease. PTHB1 exhibited the highest protein content (13.14%, *w*/*w*), which was higher than PTHB0 (10.67%, *w*/*w*), PTHB2 (10.25%, *w*/*w*), and PTHB3 (9.20%, *w*/*w*). In contrast, the fat content showed a slight decline of (0.89%, *w*/*w*) from PTHB0 to PTHB3. Starch content increased progressively with peeling, rising by 4.80%, 7.60%, and 12.77% for PTHB1, PTHB2, and PTHB3, respectively. The β-glucan content increased from (3.92%, *w*/*w*) in PTHB0 to (4.28%, *w*/*w*), (4.48%, *w*/*w*), and (4.80%, *w*/*w*) in PTHB1, PTHB2, and PTHB3, respectively. Similarly, the polyphenol content was reduced by 41.42%, 47.60%, and 52.38% in PTHB1, PTHB2, PTHB3, respectively, indicating a substantial loss of polyphenols after peeling.

The distribution of various nutrients in highland barley is uneven across different grain layers, which consist of the outer husk (rich in insoluble fiber and polyphenols), aleurone layer (mainly protein, fat, minerals, and vitamins), germ (high in protein and fat), and endosperm (primarily starch and β-glucan) [2,21]. Protein was primarily concentrated in the aleurone layer and at the aleurone–endosperm interface [22]. The higher protein content in PTHB1 indicated that one peeling preserved the aleurone layer. The repeated removal of the outer husk led to a significant loss of dietary fiber and polyphenols, while the relative content of fat, starch, and β-glucan increased, which aligns with previous studies [23]. These findings suggested that peeling could alter the nutrient distribution in highland barley.

### 3.2. Antioxidant Activity

**Figure 2:**
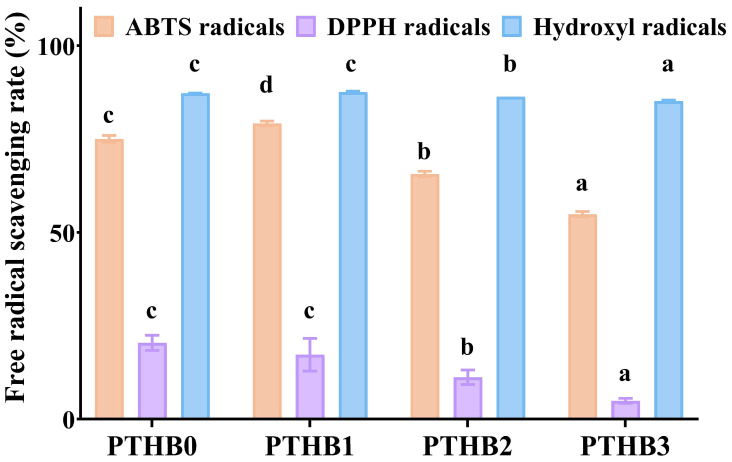
Antioxidant capacity. Unpeeled highland barley (PTHB0), Highland barley with one peeling (PTHB1), Highland barley with two peelings (PTHB2), Highland barley with three peelings (PTHB3). The different letters (a, b, c, d) in the figure indicate significant differences between groups (*p* < 0.05).

As indicated in Figure 2, highland barley with different peeling times showed potent antioxidant ability, as demonstrated by radical scavenging activity. As the number of peeling times increased, the ABTS, DPPH, and hydroxyl radical scavenging activities decreased. Compared to PTHB0, PTHB1 exhibited a 5.52% and 0.37% increase in scavenging activity against ABTS and hydroxyl radicals, respectively, while the scavenging activity against DPPH radicals decreased by 15.62%. In comparison to PTHB0, PTHB2 showed reductions in free radical scavenging activity of 12.51%, 45.05%, and 1.05% for ABTS, DPPH, and hydroxyl radicals, respectively, while PTHB3 exhibited more pronounced reductions of 26.86%, 76.02%, and 2.41%, respectively. The antioxidant capacity of highland barley was primarily attributed to its polyphenolic compounds [24]; thus, the loss of polyphenols could lead to a decrease in antioxidant activity. In addition to polyphenols, β-glucan was also reported to possess antioxidant properties. β-glucans from barley, oats, and yeast have been shown to effectively scavenge hydroxyl radicals [25]. Notably, PTHB1 exhibited the highest free radical scavenging activity, which may have been related to the exposure of the outermost layer to atmospheric oxygen, thereby affecting the bioavailability of polyphenols as a key factor influencing antioxidant activity.

### 3.3. In Vitro Starch Digestibility

**Figure 3:**
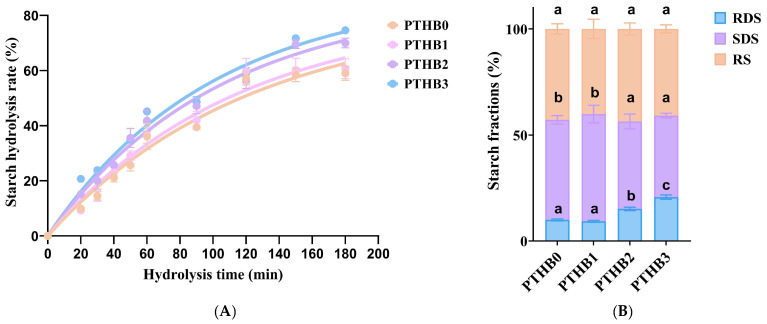
Starch hydrolysis rate (**A**), and starch fraction composition (**B**).Unpeeled highland barley (PTHB0), Highland barley with one peeling (PTHB1), Highland barley with two peelings (PTHB2), Highland barley with three peelings (PTHB3). The different letters (a, b, c) in the figure indicate significant differences between groups (*p* < 0.05).

In vitro starch digestion curves for highland barley with different peeling times are shown in Figure 3A. The hydrolysis rate of highland barley at all peeling times initially increased rapidly and then gradually slowed down over time. At 180 min, the final hydrolysis rate trends were recorded: PTHB0 < PTHB1 < PTHB2 < PTHB3. The digestion rates for PTHB1, PTHB2, and PTHB3 increased by 2.82%, 18.62%, and 26.43%, respectively, compared to PTHB0. These results suggested that increasing the number of peeling times enhanced the digestion of starch.

The final glucose concentration of the digestion reaction (*C_∞_*) and hydrolysis rate constant (*k*) are shown in Table 1. PTHB0 exhibited the lowest digestion rate (81.44%), and PTHB1 had the slowest digestion speed (8.22 × 10^−3^ min^−1^), while PTHB3 showed a significantly higher digestion rate (88.81%) and digestion speed (1.00 × 10^−2^ min^−1^) compared to the other samples (*p* < 0.05). Smaller *C_∞_* and *k* values indicated lower digestion rates and slower digestion speeds, suggesting stronger resistance to enzymatic hydrolysis. It is noteworthy that the in vitro *GI* value of unpeeled highland barley was 68.99, while after peeling once, twice, and three times, the *GI* values were 70.17, 73.56, and 75.38, respectively (*p* < 0.05). The rapid hydrolysis of starch was accompanied by an increase in blood glucose responses, which was consistent with changes in the digestion reaction (*C_∞_*) and hydrolysis rate constant (*k*). Overall, peeling increased the glycemic potential of highland barley. The *GI* value served as an indicator of postprandial blood glucose elevation and provided valuable dietary guidance for individuals with diabetes.

The differences in starch digestion characteristics in highland barley were likely attributed to the removal of the husk and changes in composition caused by peeling [26,27]. The bran and cell wall may have reduced the permeability of starch to amylase, thus decreasing amylase’s effectiveness [28,29]. Non-starch polysaccharides limit water availability for starch, thereby slowing starch hydrolysis by α-amylase. Moreover, phenolic compounds inhibit digestive enzyme activity by binding to their active sites [30].

The starch composition of highland barley with all peeling times is shown in Figure 3B. The content of *RDS* decreased from 9.92% in PTHB0 to 9.22% in PTHB1, while the content of *SDS* increased from 47.21% in PTHB0 to 50.63% in PTHB1. The content of *RS* decreased from 42.87% in PTHB0 to 40.15% in PTHB1, but no marked differences were observed (*p* < 0.05). Compared to PTHB0, the *RDS* content in PTHB2 and PTHB3 increased by 52.41% and 108.53%, respectively, while *SDS* decreased by 12.67% and 18.59%, with no significant change in *RS* content. PTHB3 contained the highest *RDS* content (20.69%). These results aligned with the digestion curves, further supporting the idea that the outer husk’s structure and composition exerted a stronger inhibitory effect on starch digestion.

### 3.4. Rheological Simulation 

**Figure 4:**
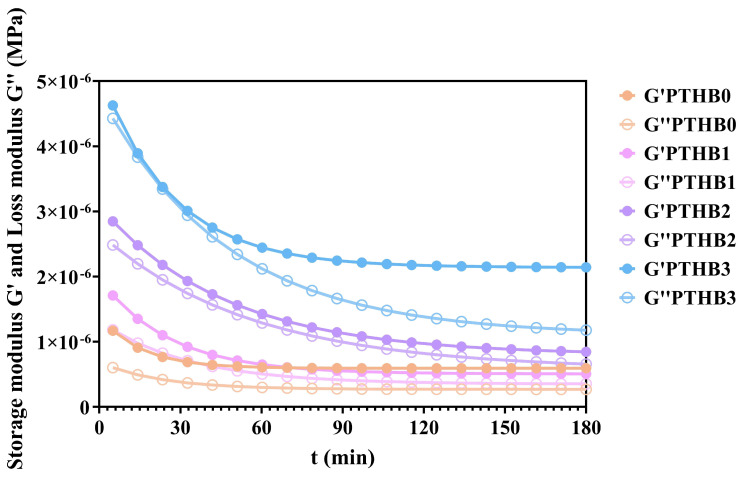
Rheological simulation of digestion.

The variations in the storage moduli (G′) and loss moduli (G″) of highland barley with different peeling times during digestion are illustrated in Figure 4. Similar to the in vitro starch hydrolysis results, the moduli showed a rapid decline in the early stage, which gradually slowed down and plateaued over time. As peeling time increased, the storage moduli decreased by 8.08 × 10^−7^, 1.03 × 10^−6^, 1.49 × 10^−6^, and 3.89 × 10^−6^, respectively, while the loss moduli decreased by 3.34 × 10^−7^, 8.12 × 10^−7^, 1.55 × 10^−6^, and 3.22 × 10^−6^, respectively, during 180 min.

The above results can be explained by the fact that during digestion, the macromolecules were broken down due to mechanical forces and enzymatic action, leading to the gradual disruption of the internal network and a significant reduction in elasticity and viscosity [31]. These findings indicated that structural degradation by digestive enzymes occurred more rapidly in samples with higher peeling times, suggesting that a higher non-starch components content and outer husk structure delayed enzymatic hydrolysis. This was consistent with the findings from the starch digestion curves.

### 3.5. Microstructure 

**Figure 5:**
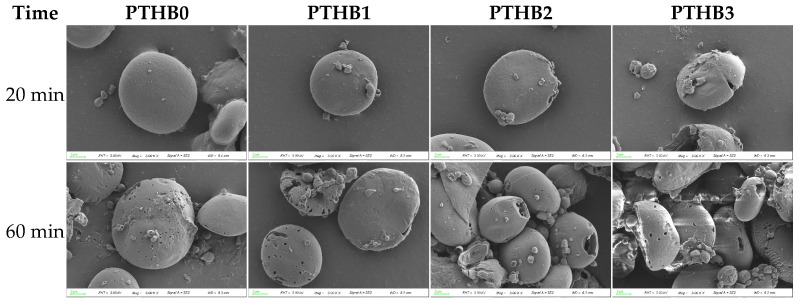
SEM at different digestion times.

The SEM images of whole barley flour granules after 20 min and 60 min of digestion with pancreatic and glucoamylase enzymes are shown in Figure 5. The starch granules exhibited an oval shape with a relatively smooth surface, and peeling did not significantly alter the granule morphology. At 20 min, PTHB0 and PTHB1 starch granules retained their original structure with no apparent changes, whereas PTHB2 and PTHB3 exhibited noticeable fissures. By 60 min, the samples with a higher number of peeling times (PTHB2 and PTHB3) lost their granular integrity, exposing internal multi-layered structures, while PTHB0 and PTHB1 largely maintained their original form, showing only smaller and shallower surface pores.

SEM images provided a more direct observation of the starch digestion process. As digestion time increased, enzymatic hydrolysis progressively degraded the starch granules, which corresponded with the findings of Yang et al. [32]. At the same digestion time, starch degradation was less pronounced with fewer peeling times. This was likely due to the protective effect of the outer husk structure and non-starch components [33]. This further supported the results of starch digestion.

### 3.6. Hypoglycemic Activity

#### 3.6.1. Fasting Blood Glucose in Mice

**Figure 6:**
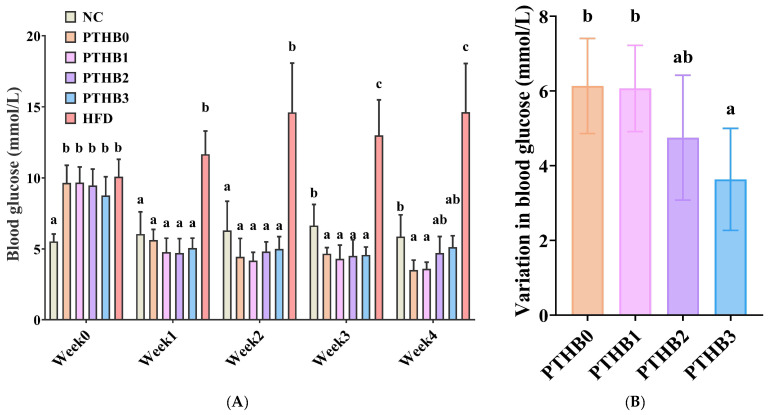
Fasting blood glucose levels (**A**), and reduction in fasting blood glucose levels (**B**). Mice were administered 100% highland barley with no peeling (PTHB0), Mice were administered 100% highland barley with one peeling (PTHB1), Mice were administered 100% highland barley with two peelings (PTHB2), Mice were administered 100% highland barley with three peelings (PTHB3). The different letters (a, b, c) in the figure indicate significant differences between groups (*p* < 0.05).

According to Figure 6A, as the intervention time increased, all highland barley samples with varying peeling times showed a notable decline in blood glucose levels in mice. Specifically, blood glucose levels decreased from 9.65 mmol/L to 3.52 mmol/L in the PTHB0 group, from 9.67 mmol/L to 3.60 mmol/L in the PTHB1 group, from 9.47 mmol/L to 4.72 mmol/L in the PTHB2 group, and from 8.77 mmol/L to 5.13 mmol/L in the PTHB3 group, respectively. These findings suggested that all highland barley samples exhibited hypoglycemic effects.

To further explore the differences in hypoglycemic effects among barley samples with different peeling times, the blood glucose reduction values (Figure 6B) were calculated. The results demonstrated that the PTHB0 and PTHB1 groups exhibited more potent hypoglycemic effects (6.13 mmol/L and 6.07 mmol/L), whereas the PTHB3 group showed a relatively weaker glucose-lowering capacity (3.63 mmol/L). This trend matched the findings from the in vitro starch digestion experiments (Table 1).

According to Zhou et al., highland barley could lower blood glucose levels and improve metabolic indicators in type Ⅱ diabetes patients, with dietary fiber and polyphenols being the main active components [34]. β-glucan and polyphenols inhibited the activity of α-glucosidase and α-amylase, which helped reduce the rate of starch digestion and lowered blood glucose levels [35]. The findings of this study further corroborated the beneficial role of the outer layers, rich in dietary fiber and polyphenols, in regulating blood glucose levels.

#### 3.6.2. Gut Microbiota in Mice

**Figure 7:**
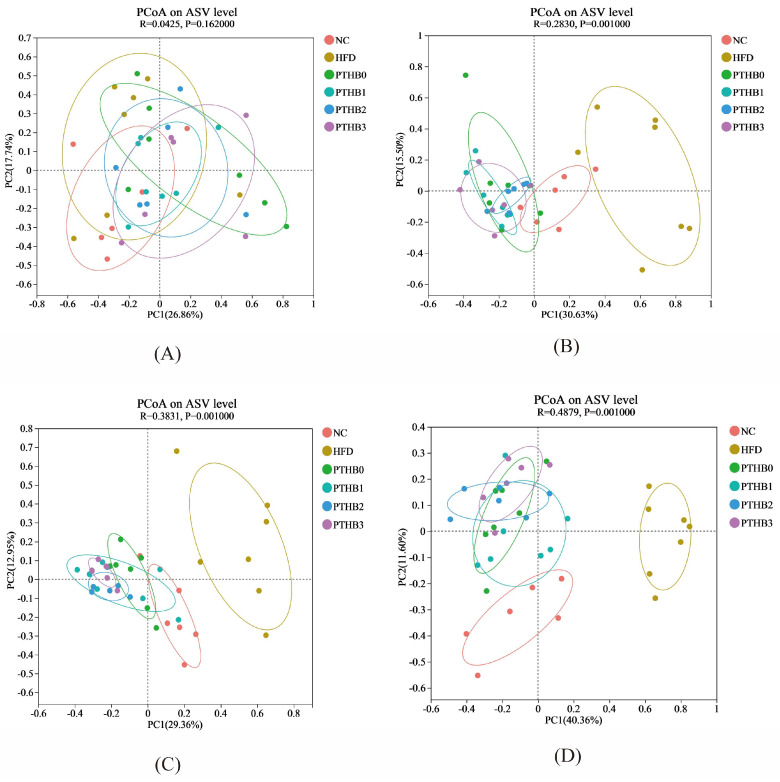
PCoA in week 1 (**A**), PCoA in week 2 (**B**), PCoA in week 3 (**C**), PCoA in week 4 (**D**).

Principal coordinate analysis (PCoA) was applied to assess the similarity of gut microbiota among the high-fat diet (HFD) group, control group (NC), and highland barley dietary intervention groups. The ASV (Amplicon Sequence Variant) is a precise way of identifying tiny differences in DNA sequences from microbes. β-diversity assessment at the ASV level, based on weekly changes during the 4-week intervention, revealed significant variations in microbial community structure, as shown in Figure 7. After two weeks of intervention, the gut microbiota composition of the HFD group significantly separated from that of the highland barley intervention groups, and the separation became more pronounced as the intervention progressed. This indicated that the 4-week highland barley dietary intervention significantly altered the gut microbiota composition with dysregulated glucose and lipid metabolism. The control group (NC) and high-fat diet (HFD) group showed increasing separation in microbial community structure over the weeks. In contrast, the control group (NC) exhibited a more similar community structure to the highland barley dietary intervention groups, indicating that highland barley intervention effectively improved the composition and abundance of the gut microbiota.

**Figure 8:**
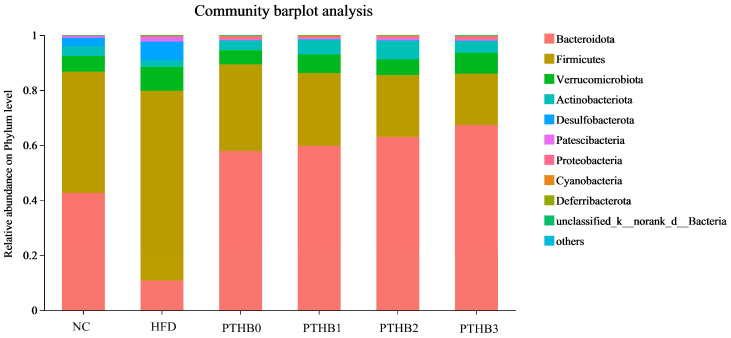
Bars chart showing the phylum level.

At the phylum level, *Firmicutes* and *Bacteroidetes* were the most prevalent phyla across all groups, as shown in Figure 8. The highland barley dietary intervention, regardless of peeling times, was able to reverse the imbalance in the *Firmicutes*/*Bacteroidetes* ratio caused by HFD/STZ. Specifically, after 4 weeks of intervention, the *Firmicutes*/*Bacteroidetes* ratio in the PTHB0 (0.54), PTHB1 (0.44), PTHB2 (0.36), and PTHB3 (0.28) groups was significantly reduced compared to the HFD/STZ group (6.40) (*p* < 0.05). As the number of peeling times decreased, the values of the peeling groups became closer to that of the NC group (1.03).

**Figure 9:**
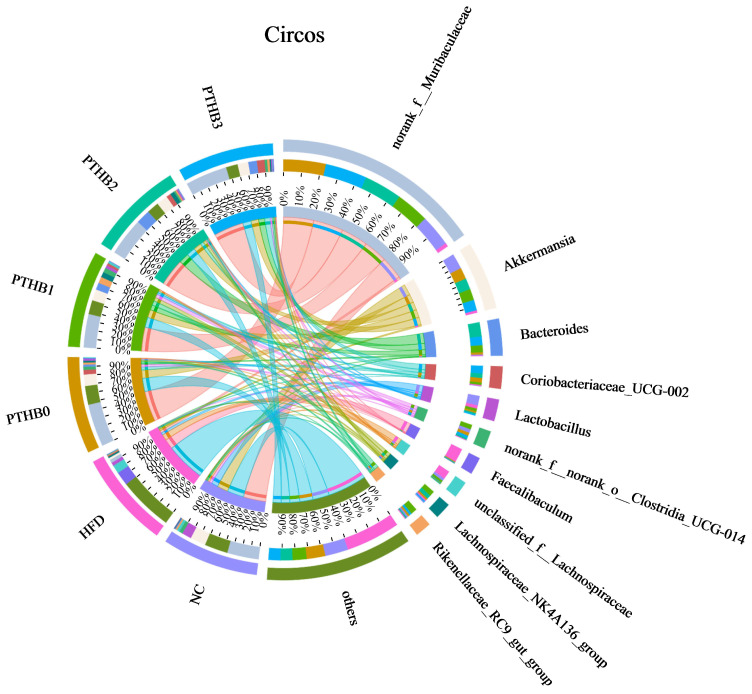
Circos on genus level.

At the genus level, compared to the sustained HFD, the highland barley dietary intervention enriched various probiotics, such as *Akkermansia*, *Lactobacillus*, and *Lachnospiraceae-NK4A136-group*, while reducing the abundance of *Faecalibaculum*, which was significantly increased in the high-fat and sugar diets [36]. *Lachnospiraceae_NK4A136_group* is a genus closely associated with short-chain fatty acids and improved metabolism [37]. Notably, *Akkermansia* is a bacterium related to glucose and lipid metabolism. Many studies have proven the effectiveness of supplementing with *Akkermansia* to lower blood glucose and lipids [38]. It can reduce carbohydrate absorption to decrease energy intake and indirectly stimulate insulin secretion. *Lactobacillus* helps preserve regulate glucose balance and intestinal barrier integrity by stimulating GLP-1 secretion [39]. According to Figure 9, the abundance of *Akkermansia* and *Lactobacillus* was inversely correlated with the number of peeling times, which might account for the differences in the hypoglycemic activity.

Previous studies have emphasized that the gut microbiota plays a crucial role in host metabolic regulation, including glucose metabolism, secondary metabolite synthesis, and bile acid metabolism [6,40]. Non-digestible bioactive components, such as dietary fiber and polyphenols, reach the colon intact, where they serve as substrates for the gut microbiota, thereby influencing the composition and function of microbial community. The effects on the *Firmicutes*/*Bacteroidetes* ratio and the enrichment of beneficial genera varied with peeling times. These findings suggested that the utilization of highland barley by the gut microbiota was dependent on the peeling times. Fewer peeling times were more beneficial for increasing the hypoglycemic activity of highland barley.

## 4. Conclusions

This study systematically analyzed the effects of different peeling times on the nutritional components, digestive properties, and blood glucose-lowering activity of highland barley. The results showed that with increasing peeling times, the digestibility and digestion speed of highland barley increased, accompanied by a higher proportion of rapidly digestible starch. The retention of outer structures rich in dietary fiber, protein, and polyphenols delayed enzyme accessibility to the digestive substrates, thereby slowing down starch digestion in highland barley. We also assessed the impact of different peeling times on blood glucose regulation in mice with dysregulated glucose and lipid metabolism induced by the HFD/STZ method. The results aligned with the in vitro digestion findings, emphasizing the potential of low peeling times in regulating blood glucose. We further investigated the impact of peeling on the distal digestive tract, revealing that the highland barley dietary intervention reversed the imbalance in the *Firmicutes*/*Bacteroidetes* ratio induced by HFD/STZ and enriched the content of probiotics. Additionally, the application of highland barley with fewer peeling times demonstrated a more pronounced enrichment of *Akkermansia* and *Lactobacillus*.

By studying highland barley with different component compositions generated through peeling treatments, this research provides a scientific basis for optimizing the techniques used to process highland barley, aiming to maximize its potential as a functional food. At the same time, this study offers promising dietary strategies for the management of metabolic diseases such as diabetes and obesity, as well as for improving metabolic health. Future research should investigate the contributions of various components and their relative proportions to the blood glucose-lowering effects of highland barley. Moreover, it is essential to explore the specific mechanisms behind the compositional changes induced by peeling in the complex metabolic environment in vivo, along with their long-term impact. Understanding this will be key to advancing the use of highland barley to treat metabolic diseases. Additionally, the practical application of highland barley in functional cereal foods should be a primary focus of future studies.

## Figures and Tables

**Table 1:** In vitro digestion first-order kinetic fitting parameters and glycemic index. The final glucose concentration of the digestion reaction (*C_∞_*) and hydrolysis rate constant (*k*), and glycemic index (*GI*). The different letters (a, b, c) in the figure indicate significant differences between groups (*p* < 0.05).

Sample	*C_∞_* (%)	*k* (10^−3^ min^−1^)	*GI*
PTHB0	81.44 ± 4.27 ^a^	8.22 ± 0.92 ^a^	68.99 ± 1.41 ^a^
PTHB1	81.62 ± 4.80 ^a^	8.71 ± 0.18 ^ab^	70.17 ± 2.14 ^a^
PTHB2	86.20 ± 0.07 ^ab^	9.67 ± 0.70 ^bc^	73.56 ± 1.36 ^b^
PTHB3	88.81 ± 1.80 ^b^	10.04 ± 0.62 ^c^	75.38 ± 0.67 ^b^

## Data Availability

The original contributions presented in this study are included in the article. Further inquiries can be directed to the corresponding author.

## Abbreviations

PTHB0	Unpeeled highland barley
PTHB1	Highland barley with one peeling
PTHB2	Highland barley with two peelings
PTHB3	Highland barley with three peelings
